# Effect of Acrylamide Treatment on Cyp2e1 Expression and Redox Status in Rat Hepatocytes

**DOI:** 10.3390/ijms23116062

**Published:** 2022-05-28

**Authors:** Jelena Marković Filipović, Marko Miler, Danijela Kojić, Jelena Karan, Ivana Ivelja, Jovana Čukuranović Kokoris, Milica Matavulj

**Affiliations:** 1 Department of Biology and Ecology, Faculty of Sciences, University of Novi Sad, Trg Dositeja Obradovića 2, 21000 Novi Sad, Serbia; danijela.kojic@dbe.uns.ac.rs (D.K.); jelena.karan@dbe.uns.ac.rs (J.K.); ivana.ivelja@dbe.uns.ac.rs (I.I.); milica.matavulj@dbe.uns.ac.rs (M.M.); 2 Department of Cytology, Institute for Biological Research “Siniša Stanković”-National Institute of Republic of Serbia, University of Belgrade, Bulevar despota Stefana 142, 11060 Belgrade, Serbia; marko.miler@ibiss.bg.ac.rs; 3 Department of Anatomy, Faculty of Medicine, University of Niš, Blvd. Dr Zorana Djindjica 81, 18000 Niš, Serbia; jovana.cukuranovic.kokoris@medfak.ni.ac.rs

**Keywords:** acrylamide, hepatocyte, redox status, cytochrome P450 2E1, immunohistochemistry, rat, H4IIE cell line, in vitro, in vivo

## Abstract

Acrylamide (AA) toxicity is associated with oxidative stress. During detoxification, AA is either coupled to gluthatione or biotransformed to glycidamide by the enzyme cytochrome P450 2E1 (CYP2E1). The aim of our study was to examine the hepatotoxicity of AA in vivo and in vitro. Thirty male Wistar rats were treated with 25 or 50 mg/kg b.w. of AA for 3 weeks. Qualitative and quantitative immunohistochemical evaluation of inducible nitric oxide synthase (iNOS), CYP2E1, catalase (CAT), superoxide dismutase 1 (SOD1), and SOD2 expression in liver was carried out. Bearing in mind that the liver is consisted mainly of hepatocytes, in a parallel study, we used the rat hepatoma cell line H4IIE to investigate the effects of AA at IC_20_ and IC_50_ concentrations on the redox status and the activity of CAT, SOD, and glutathione-S-transferase (GST), their gene expression, and CYP2E1 and iNOS expression. Immunohistochemically stained liver sections showed that treatment with AA_25mg_ induced a significant decrease of CYP2E1 protein expression (*p* < 0.05), while treatment with AA_50mg_ led to a significant increase of iNOS protein expression (*p* < 0.05). AA treatment dose-dependently elevated SOD2 protein expression (*p* < 0.05), while SOD1 protein expression was significantly increased only at AA_50mg_ (*p* < 0.05). CAT protein expression was not significantly affected by AA treatments (*p* > 0.05). In AA-treated H4IIE cells, a concentration-dependent significant increase in lipid peroxidation and nitrite levels was observed (*p* < 0.05), while GSH content and SOD activity significantly decreased in a concentration-dependent manner (*p* < 0.05). AA IC_50_ significantly enhanced GST activity (*p* < 0.05). The level of mRNA significantly increased in a concentration-dependent manner for iNOS, SOD2, and CAT in AA-treated H4IIE cells (*p* < 0.05). AA IC_50_ significantly increased the transcription of SOD1, GSTA2, and GSTP1 genes (*p* < 0.05), while AA IC_20_ significantly decreased mRNA for CYP2E1 in H4IIE cells (*p* < 0.05). Obtained results indicate that AA treatments, both in vivo and in vitro, change hepatocytes; drug-metabolizing potential and disturb its redox status.

## 1. Introduction

Acrylamide (AA) is an important industrial chemical that has been available commercially since the mid-1950s [1]. A non-toxic polymer of acrylamide, polyacrylamide (PAA), has many applications in industry. PAA is used in the paper packaging, dye synthesis, pesticides’ production, soil stabilizing, cement production, textile, and cosmetic industries and as flocculant for waste water treatments [2]. Additionally, in molecular biology, PAA gels are used for DNA and protein electrophoresis [3]. Although PAA is non-toxic, photochemical reactions and high temperatures could induce its releasing of residual amounts of AA monomer, and thus contributes to the contamination of the environment [4]. However, the major source of AA exposure in the general human population is dietary acrylamide. AA is produced in plant food rich in carbohydrates and low in proteins, when it is prepared at high temperature (higher than 120 °C), such as frying and baking [5]. AA is a Maillard reaction product that is formed during the heat processing of starchy food that contains reduced sugars and the amino acid asparagin. High temperatures and lack of water during food processing promote AA formation [6]. Therefore, AA has been detected in food prepared by heating/frying in a pan, in an oven, or a microwave oven, but was not found in the food prepared by boiling [7,8]. In the modern diet, the major sources of AA intake are fried potato products, bread, cookies, crackers, and breakfast cereals [9]. Regulatory agencies have not established mandatory limits for the maximum acceptable AA levels in food. However, they provided recommendations for the reduction of AA formation in food [10]. Namely, in 2009, the FAO/WHO Codex Alimentarius developed a “Code of Practice for the Reduction of Acrylamide in Foods (CAC/RCP 67-2009)”, which provides guidance for the prevention and reduction of AA formation in potato and cereal products [10,11]. In 2015, the Spanish Agency for Consumer Affairs, Food Safety, and Nutrition (AECOSAN) established recommendations for reducing AA formation during the thermal processing of food [10,12]. They recommended preparing food in a microwave oven or baking instead of frying, reducing the time and temperature of food frying (<175 °C), and avoiding the reuse of frying oil. In 2016, the Food and Drug Administration (USA) developed a “Guide for industry” that defined strategies for the reduction of AA formation in food, dedicated to food producers, manufacturers, and operators [10,13]. In 2017, the European Union issued the “Commission Regulation (EU) 2017/2158” establishing mitigation measures and benchmark levels for the reduction of the presence of AA in food [10,14]. Finally, in 2019, the confederation of the food industry in the European Union FoodDrinkEurope defined various intervention steps in order to prevent and reduce AA formation in specific products [10,15]. According to the physiologically based toxicokinetic (PBPK or PBTK) model, the tolerable daily intake for AA neurotoxicity and cancer is estimated to be 40 and 2.6 µg/kg-day, respectively [16].

Neurotoxicity, genotoxicity, and carcinogenicity, as well as the reproductive, and developmental toxicity of AA have been detected in in vivo and in vitro model systems [17,18]. According to the International Agency for Research on Cancer (IARC), AA is classified as a putative human carcinogen [19]. Bearing in mind AA’s toxicity, and that thermal processing is an indispensable way for food preparation in the contemporary diet, AA occurrence in frequently consumed food items provokes significant public concern.

In the liver, during the detoxification process, AA is either coupled to gluthatione (GSH) or metabolized to more toxic glycidamide (GA) by the enzyme cytochrome P450 2E1 (CYP2E1) [20]. GA is genotoxic in both in in vitro and in vivo model systems, and can form DNA and hemoglobin adducts [1]. 

AA exerts toxic effects via oxidative stress induction. Oxidative stress is defined as an imbalance between the systematic impairment triggered by reactive oxygen species (ROS) and/or reactive nitrogen species (RNS) and the physiological ability to readily neutralize the deleterious toxic intermediates [18]. Impaired redox status may lead to cytotoxicity and genotoxicity [21]. 

Bearing in mind that toxic AA is metabolized in the liver, and that oxidative stress is a main mechanism of AA-induced toxicity, the aim of our research was to investigate the effects of AA on oxidative stress parameters in the liver in in vivo and in vitro model systems. In addition, in both model systems, we analyzed the expression of the enzyme responsible for AA biotransformation—CYP2E1. Since the liver consists of 80% parenchymal cells, i.e., hepatocytes, and 20% non-parenchymal cells [22], we used the rat hepatoma cell line—H4IIE—to analyze the hepatotoxic potential of AA in vitro. H4IIE is a validated model system for the evaluation of toxicity and expression of cytochrome *P450* enzymes [23,24]. In our study, we examined the effects of AA on the expression of inducible *nitric oxide synthase* (iNOS), catalase (CAT), superoxide dismutase 1 (SOD1), SOD2, and CYP2E1 in vivo in rat liver and in vitro in H4IIE cells. In addition, we investigated effects of AA on oxidant and antioxidant biomarkers in the H4IIE cell line.

## 2. Material and Methods

### 2.1. Animals and Treatment

The study was conducted on adult male Wistar rats (*Rattus norvegicus*) that were 65 days old at the beginning of the experiments. Rats were housed under controlled laboratory conditions (22–24 °C; 12 h night: 12 h light) and given standard granulated rodent food and tap water *ad libitum*. Rats were randomly allocated into three groups (*n* = 10).

The level of AA in carbohydrate-rich food is approximately 2300 µg/kg, while the daily exposure of a 75 kg adult is estimated to be 61 µg/kg b.w. [25,26]. According to the dose conversion relation between rodents and humans, rodents’ daily intake dose is approximately 500 µg/kg b.w., which corresponds to 50 mg/kg b.w. for short-time exposure [26]. According to the OECD protocol for dosage setting in subchronic studies, two-fold intervals are optimal for descending dose levels [27]. Therefore, acrylamide (Sigma Aldrich) was applied in doses of 25 or 50 mg/kg b.w. by oral gavage for 21 days, while the control group received vehicle (distilled water) orally. Selection of the doses applied in this study was made according to other studies concerning AA subchronic treatment [2,28,29]. After treatment termination, AA-exposed and control rats were sacrificed by decapitation under diethyl ether anesthesia.

The experimental procedures were in accordance with the Directive 2010/63/EU on the protection of animals used for experimental and other scientific purposes and were approved by the Ethical Committee on Animal Experiments of the University of Novi Sad (No. I-2011-03).

### 2.2. Organ Processing and Immunohistochemical (IHC) Staining Procedures

The liver sample was taken from the middle lobe, fixed in 10% formalin, standardly prepared for embedding in paraffin, and cut into 5 μm thick paraffin sections. For analysis of iNOS, CYP2E1, SOD1, SOD2, and CAT expression, liver sections were stained immunohistochemically following the Ultravision LP Detection System protocol (TL-125-HD, Thermo Scientific) according to manufacturer’s instructions as described previously [2,29]. IHC was carried out using an anti-SOD1 antibody (1:1000, ab13498, Abcam), anti-SOD2 antibody (1:200, ab13533, Abcam), anti-CAT antibody (1:1000, ab16731, Abcam), anti-CYP2E1 antibody (1:200, ab84598, Abcam), and anti-iNOS antibody (1:100, ab15323, Abcam).

Digital images of stained sections were taken on a Motic™ B3 Series microscope with Moticam 2500 camera (Motic).

### 2.3. Quantitative Analyses of Digital Immunohistochemistry Images

Quantitative analyses of digital images were performed using the ImageJ program (Image J, Version 1.50f) as described in Marković et al. [29] and Stošić et al. [2], according to the protocols of Ruifrok and Johnston [30] and Varghese et al. [31]. Briefly, color deconvolution using the DAB vector for IHC-stained sections was applied for the separation of the color spectra. Optical density (OD) was determined since OD corresponds to the amount of the stain. The OD was calculated as:

OD = −log10 (I_C_/I_0·C_),

where I_C_ represents the intensity of the detected light after passing through the *specimen* and I_0·C_ is the intensity of light entering the specimen.

Stained percentage color area was determined using ImageJ plugin—IHC profiler as reported previously by Varghese et al. [31].

In order to quantify staining intensity, 40 unbiasedly taken digital images of IHC-stained liver sections of each rat were examined.

### 2.4. Cell Culture and Treatment

The rat hepatoma cell line (H4IIE) (ATCC^®^ CRL-1548™) was grown in MEM supplemented with 10% FBS and penicillin/streptomycin pH 7.4, at 37 °C in a humidified atmosphere containing 5% CO_2_. Cell culture reagents were purchased from Gibco (Paisley). Cells were treated with 4 mM AA (IC_20_) and 4.5 mM (IC_50_) for 24 h.

### 2.5. Cell Viability Assays

H4IIE cells’ viability was assessed by the MTT (3-(4,5-Dimethylthiazol-2-yl)-2,5-diphenyl tetrazolium bromide) and trypan blue viability assays as described in Marković et al. [29]. For the MTT viability assay cells were cultivated in a 96-well plate and exposed to increasing concentrations of AA (2.5–5.5 mM) for 24 h. Cell viability was expressed in percentages after comparing with control non-treated cells that were presumed to be 100% viable. For the trypan blue viability assay, cells were cultivated in a 6-well plate. After AA application, cells were collected by trypsinization and mixed with sterile 0.4% trypan blue solution. The numbers of dead and alive cells were determined by microscopy using a hemocytometer. Both assays were carried out in six replicates and repeated three times.

### 2.6. Evaluation of Redox Status and Glutathione-S-Transferase (GST) Activity

Protocols for the determination of reduced glutathione (GSH), protein thiol groups (SH) concentrations, NO production, lipid peroxidation, SOD, CAT, and GST activities were described previously in Marković et al. [29]. Protein concentration was measured by the Bradford method [32], while bovine serum albumin (Bio-Rad) served as a protein standard. All the assays were carried out in three biological replicants.

### 2.7. RNA Isolation and Real-Time RT-PCR (RT-qPCR)

Total RNA from H4IIE control and AA-treated cells was isolated with the RNAqueous^®^-4PCR Kit (Applied Biosystems). RNA concentration was determined using a BioSpec-nano spectrophotometer (Shimadzu). cDNA synthesis was performed using a High Capacity cDNA Reverse Transcription Kit (Applied Biosystems), while for RT-qPCR, a Power SYBR^®^ Green PCR Master Mix (Applied Biosystems) was used. mRNA levels were measured with a real-time PCR machine MasterCycler RealPlex 4 (Eppendorf). The amplification program comprised an initial denaturation step at 95 °C for 10 min and 40 cycles of a 2-step PCR program at 95 °C for 15 s and 60 °C for 1 min. The primer sequences were: *CAT*-f: 5′-CCAGCGACCAGATGAAGCA-3′; *CAT*-r: 5′-TGGTCAGGACATCGGGTTTC-3′; *SOD1*-f: 5′-AAGCGGTGAACCAGTTGTG-3′; *SOD1*-r: 5′-CCAGGTCTCCAACATGCC-3′; *SOD2*-f: 5′-GGTGGAGAACCCAAAGGAGA-3′; *SOD2*-r: 5′-AGCAGTGGAATAAGGCCTGT-3′; iNOS-f: 5′-TGCTAATGCGGAAGGTCATG-3′; iNOS-r: 5′-GCTTCCGACTTTCCTGTCTCA-3′; GSTP1-f: 5′-GGCATCTGAAGCCTTTTGAG-3′; GSTP1-r: 5′-CGAGAGACGGATACACCGAG-3′; GSTA2-f: 5′-CCCAATGTGAAGAAGTTCCTG-3′; GSTA2-r: 5′-AATTGGACAGTGCAGCTCCGCTAA-3′; CYP2E1-f: 5′-TCCCCAAGTCTTTCACCAAGTT-3′; CYP2E1-r: 5′-GAGCCAAGGTGCAGTGTGAAC-3′; beta-actin-f: 5′-AGATTACTGCCCTGGCTCCT-3′; beta-actin-r: 5′-ACATCTGCTGGAAGGTGGAC-3′. Negative controls without a template were used in all RT-qPCR reactions. The expression levels of examined genes were related to the averaged expression level of beta-actin as the housekeeping gene. All of the results were obtained from three experiments carried out in triplicate.

### 2.8. Statistical Analysis

The program STATISTICA^®^ version 13.0 (StatSoft, Inc) was used for statistical analysis. Obtained results are represented as means ± standard error of mean. For comparing differences between means, the one-way ANOVA followed by the Bonferroni’s post hoc test was applied. Values of *p* less than 0.05 were considered statistically significant.

## 3. Results

### 3.1. Cell Viability Analysis

**Figure 1:**
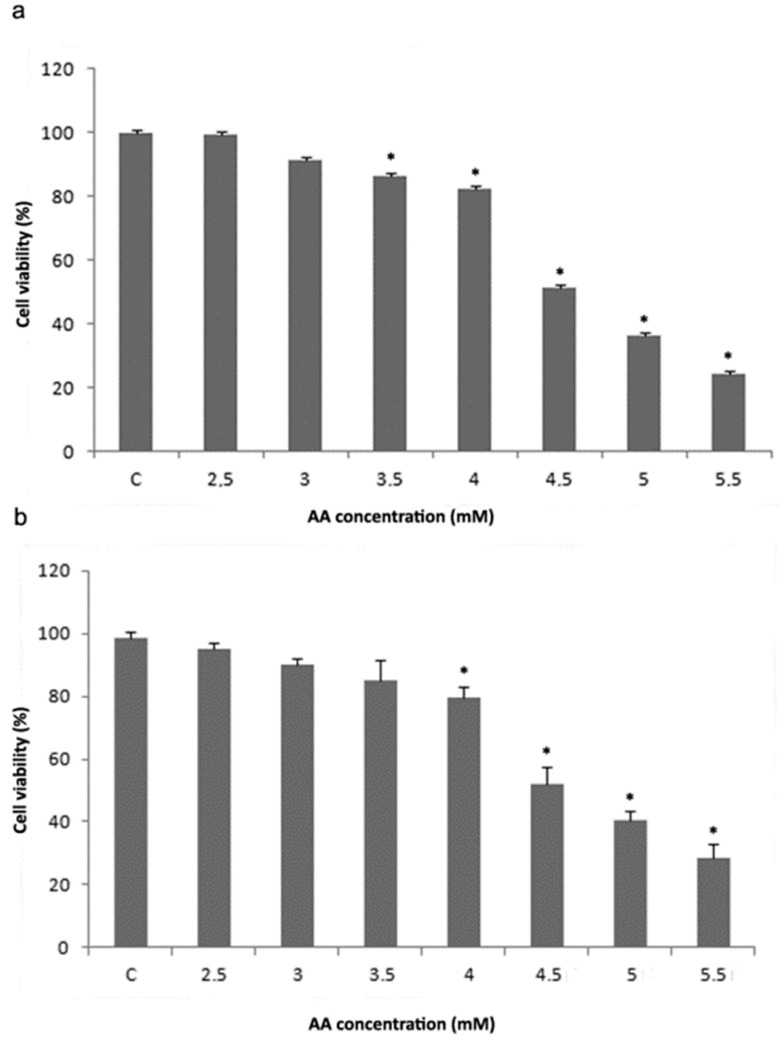
MTT (**a**) and trypan blue (**b**) viability assays performed on H4IIE cells after treatment with increasing concentrations of acrylamide (AA). Values in charts are means ± SEM of three experiments performed in six replicates. Mean values were significantly different from that of untreated control cells (* *p* < 0.05).

To investigate the toxicity of AA on hepatocytes, liver parenchymal cells, we used the rat hepatocyte cell line, H4IIE, as a model system. After treatment of H4IIE cells with increasing concentrations of acrylamide for 24 h, using MTT and trypan blue tests we determined cell viability. According to both assays, exposure to AA reduced the viability of H4IIE cells in a concentration-dependent manner (Figure 1a,b). The MTT assay revealed a significant viability reduction after treatment of the cells with 3.5, 4, 4.5, 5, and 5.5 mM acrylamide (Figure 1a), while the trypan blue assay showed that AA concentrations of 4, 4.5, 5, and 5.5 mM induced a significant viability decrease (Figure 1b). According to MTT and trypan blue viability assays, an AA concentration of 4 mM induced death of 17.62 and 20.33% H4IIE cells, respectively. Therefore, the AA concentration of 4 mM was taken as the IC_20_ in further in vitro experiments. In addition, MTT and trypan blue viability assays showed that an AA concentration of 4.5 mM caused the death of 48.45 and 48.2% cells, respectively indicating that 4.5 mM AA is the IC_50_.

### 3.2. Analysis of Redox Status and GST Activity

**Figure 2:**
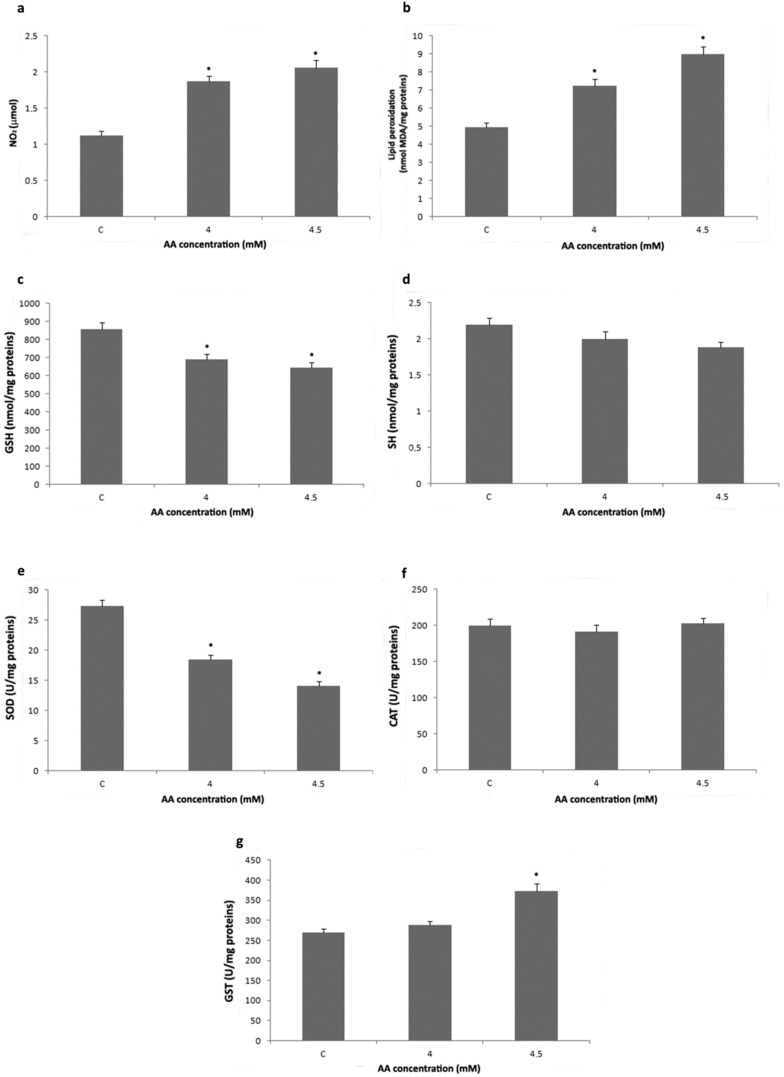
Nitrite concentration (**a**), malondialdehyde (MDA) concentration (**b**), reduced glutathione (GSH) concentration (**c**), protein thiol groups (SH) concentration (**d**), total superoxide dismutase (SOD) activity (**e**), catalase (CAT) activity (**f**), and total glutathione-S-transferase (GST) activity (**g**) in H4IIE cells after treatment with 4 and 4.5 mM acrylamide (AA) for 24 h. Values in charts are means ± SEM of three experiments performed in triplicate. Mean values were significantly different from that of untreated control cells (* *p* < 0.05).

To investigate the effects of AA on oxidant parameters and the activity of GST and antioxidant enzymes. H4IIE cells were exposed to 4 mM (IC_20_) and 4.5 mM (IC_50_) AA for 24 h. Exposure to AA caused a significant concentration-dependent increase of nitrite levels and lipid peroxidation in H4IIE cells (Figure 2a,b). On the other hand, GSH content and total SOD activity significantly decreased in a concentration-dependent manner (Figure 2c,e). Treatment with 4.5 mM AA significantly increased total GST activity (Figure 2g), while there was no significant difference in protein thiol groups level and CAT activity between control and AA-treated cells (Figure 2d,f).

**Figure 3:**
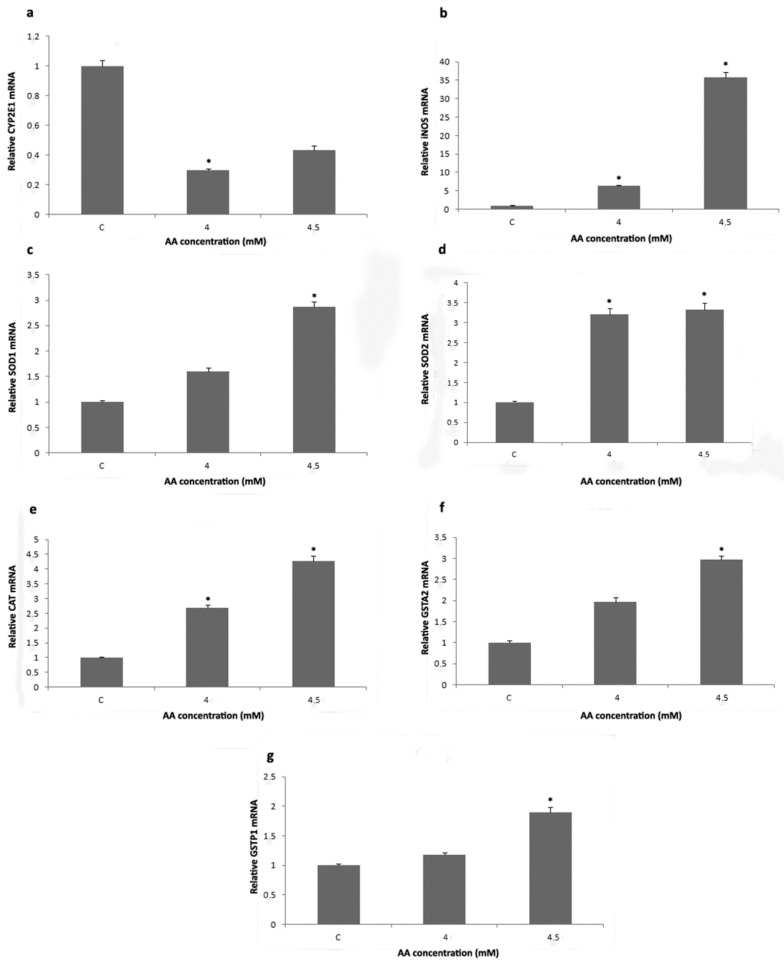
Relative transcription of cytochrome P450 2E1 (CYP2E1) (**a**), inducible *nitric oxide synthase* (iNOS) (**b**), superoxide dismutase 1 (SOD1) (**c**), superoxide dismutase 2 (SOD2) (**d**), catalase (CAT) (**e**), glutathione S-transferase alpha 2 (GSTA2) (**f**), and glutathione S-transferase pi 1 (GSTP1) (**g**) genes in H4IIE cells after treatment with 4 and 4.5 mM acrylamide (AA) for 24 h. Values in charts are means ± SEM of three experiments performed in triplicate. Mean values were significantly different from those of untreated control cells (* *p* < 0.05).

Estimation of the amount of transcribed CYP2E1 revealed that the exposure of H4IIE cells to 4 mM AA significantly decreased CYP2E1 mRNA level (Figure 3a). The level of CYP2E1 mRNA in H4IIE cells treated with 4.5 mM AA was also decreased in comparison to CYP2E1 mRNA level in control cells, but without statistical significance (Figure 3a). After AA treatments, the mRNA level for iNOS, SOD2, and CAT was significantly elevated in a concentration-dependent manner (Figure 3b,d,e). Treatment with a higher AA concentration (4.5 mM) significantly increased the transcription of SOD1, GSTA2, and GSTP1 genes (Figure 3c,f,g).

### 3.3. Immunihistochemical Analysis

**Figure 4:**
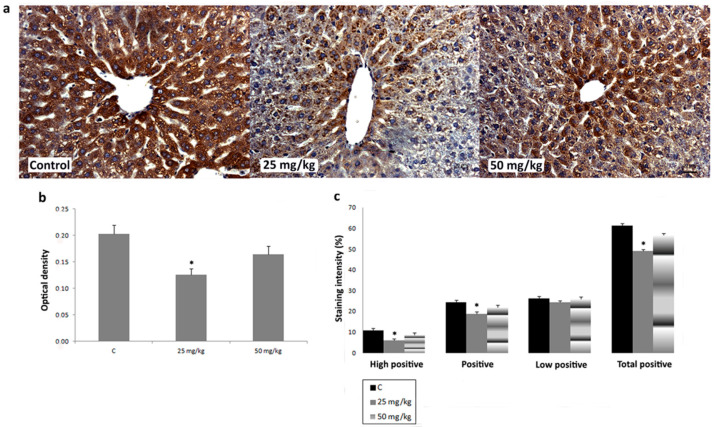
Representative micrographs of cytochrome P450 2E1 (CYP2E1) immunohistochemical staining in liver of control rats, rats treated with acrylamide (AA) in dose of 25 mg/kg b.w., and rats treated with acrylamide in dose of 50 mg/kg b.w. (**a**). Scale bar 20 μm. Optical density of CYP2E1 immunopositive cells in control and AA-treated rats in doses of 25 and 50 mg/kg b.w. (**b**). Percentage contribution of high positive, positive, low positive, and total positive immunohistochemical staining of CYP2E1 in control and AA-treated rats in doses of 25 and 50 mg/kg b.w. (**c**). Values in charts are means ± SEM; *n* = 10, * *p* < 0.05. In statistical analysis, AA-treated animals were compared with the control group.

**Figure 5:**
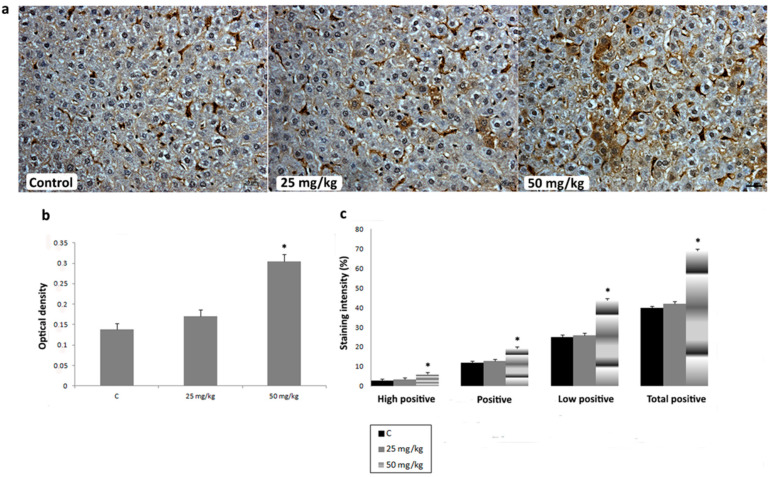
Representative micrographs of inducible nitric oxide synthase (iNOS) immunohistochemical staining in liver of control rats, rats treated with acrylamide (AA) in dose of 25 mg/kg b.w., and rats treated with acrylamide in dose of 50 mg/kg b.w. (**a**). Scale bar 20 μm. Optical density of iNOS immunopositive cells in control and AA-treated rats in doses of 25 and 50 mg/kg b.w. (**b**). Percentage contribution of high positive, positive, low positive, and total positive immunohistochemical staining of iNOS in control and AA-treated rats in doses of 25 and 50 mg/kg b.w. (**c**). Values in charts are means ± SEM; *n* = 10, * *p* < 0.05. In statistical analysis, the AA-treated animals were compared with the control group.

**Figure 6:**
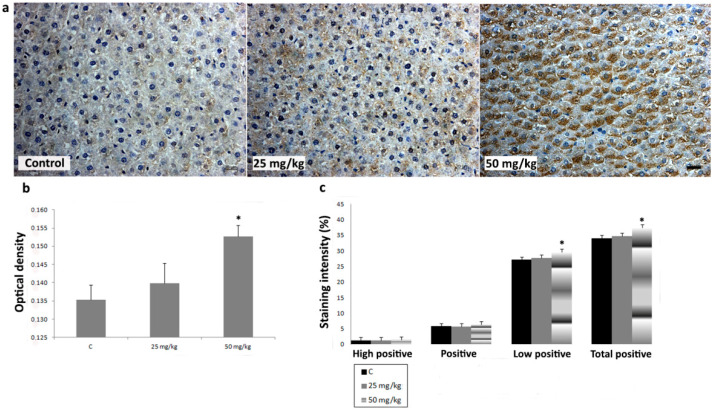
Representative micrographs of superoxide dismutase 1 (SOD1) immunohistochemical staining in liver of control rats, rats treated with acrylamide (AA) in dose of 25 mg/kg b.w., and rats treated with acrylamide in dose of 50 mg/kg b.w. (**a**). Scale bar 20 μm. Optical density of SOD1 immunopositive cells in control and AA-treated rats in doses of 25 and 50 mg/kg b.w. (**b**). Percentage contribution of high positive, positive, low positive, and total positive immunohistochemical staining of SOD1 in control and AA-treated rats in doses of 25 and 50 mg/kg b.w. (**c**). Values in charts are means ± SEM; *n* = 10, * *p* < 0.05. In statistical analysis, the AA-treated animals were compared with the control group.

**Figure 7:**
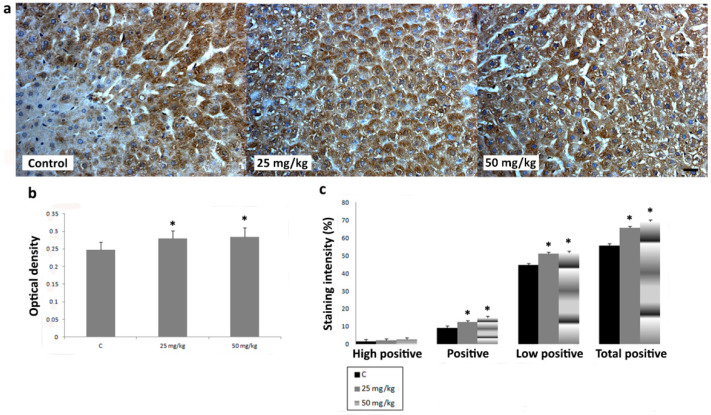
Representative micrographs of superoxide dismutase 2 (SOD2) immunohistochemical staining in liver of control rats, rats treated with acrylamide (AA) in dose of 25 mg/kg b.w., and rats treated with acrylamide in dose of 50 mg/kg b.w. (**a**). Scale bar 20 μm. Optical density of SOD2 immunopositive cells in control and AA-treated rats in doses of 25 and 50 mg/kg b.w. (**b**). Percentage contribution of high positive, positive, low positive, and total positive immunohistochemical staining of SOD2 in control and AA-treated rats in doses of 25 and 50 mg/kg b.w. (**c**). Values in charts are means ± SEM; *n* = 10, * *p* < 0.05. In statistical analysis, the AA-treated animals were compared with the control group.

**Figure 8:**
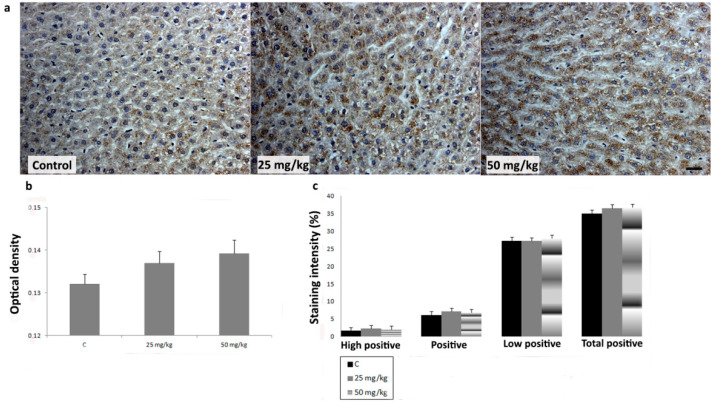
Representative micrographs of catalase (CAT) immunohistochemical staining in the liver of control rats, rats treated with acrylamide (AA) in dose of 25 mg/kg b.w., and rats treated with acrylamide in dose of 50 mg/kg b.w. (**a**). Scale bar 20 μm. Optical density of CAT immunopositive cells in control and AA-treated rats in doses of 25 and 50 mg/kg b.w. (**b**). Percentage contribution of high positive, positive, low positive, and total positive immunohistochemical staining of CAT in control and AA-treated rats in doses of 25 and 50 mg/kg b.w. (**c**). Values in charts are means ± SEM; *n* = 10.

To investigate the effects of AA treatment on CYP2E1, iNOS, SOD1, SOD2, and CAT expression in liver, we analyzed IHC-stained liver sections of rats subchronically exposed to 25 or 50 mg/kg b.w. of AA. In IHC-stained liver sections of all animal groups, an intralobular gradient in the intensity of CYP2E1 immunoreactivity was detected. CYP2E1-positive hepatocytes were dominantly distributed in centrilobular regions (Figure 4a). Treatment with AA_25mg_ decreased the intensity of immunostaining (Figure 4a). This Ddetected decrease was confirmed by a significantly reduced percentage contribution of positive, high positive, and total positive cells, as well as the optical density (OD) of immunolabeled CYP2E1 (Figure 4b,c). The contribution percentage of low positive, positive, high positive, and total positive cells, as well as the OD of immunolabeled CYP2E1 in animals exposed to AA_50mg_ was also decreased when compared to the control, but without statistical significance (Figure 4b,c). In iNOS-IHC-stained liver sections of control animals iNOS immunopositivity was detected in non-parenchymal liver cells. In AA-treated animals, besides non-parenchymal liver cells, iNOS expression was detected in intense positive immunoreactivity in the cytoplasm of hepatocytes (Figure 5a). AA treatment induced a dose-dependent increase of percentage contribution of low positive, positive, high positive, and total positive cells as well as the OD of immunolabeled iNOS (Figure 5b,c). The increase of all examined parameters proved to be statistically significant for the group treated with AA_50mg_ when compared to the control (Figure 5 b,c). Immunostaining of SOD1 in liver of control rats showed weak cytoplasmic immunoreactivity in hepatocytes (Figure 6a). AA application induced a dose-dependent increase of immunostaining intensity (Figure 6a). Significant increase of the OD and percentage contribution in the low positive and total positive cells of immunostained SOD1 was detected in group treated with AA_50mg_ (Figure 6b,c). In SOD2-IHC-stained liver sections of control animals, SOD2 expression was detected as an intense positive immunoreactivity in the cytoplasm of the majority of hepatocytes (Figure 7a). AA application led to a dose-dependent increase in the staining intensity (Figure 7a) that was confirmed by significantly increased values of the OD and percentage contribution of low positive, positive, and total positive cells in AA-treated animals (Figure 7b,c). Microscopic examination of CAT-IHC-stained liver sections of all rats’ groups showed cytoplasmic localization and medium staining intensity in hepatocytes (Figure 8a). Neverthless, AA treatment slightly increased the staining intensity and the OD of CAT immunolabeled cells; statistical analysis showed that the observed increase was not significant (Figure 8b,c).

## 4. Discussion

After absorption, acrylamide is either conjugated with reduced gluthatione or oxidized to the reactive genotoxic epoxide intermediate GA. GA is highly reactive and can form GA–DNA and GA–hemoglobin adducts. It is assumed that the formation of GA–DNA adducts promotes mutagenicity, reproductive toxicity, and carcinogenicity [33]. The transformation of AA to GA is mediated by CYP2E1 in the liver through the oxygenation of the double bond [34]. Our results are in agreement with previous reports showing an intralobular gradient of CYP2E1 expression in liver. Namely, Ingelman-Sundberg et al. [35] and Tsutsumi et al. [36] detected a higher level of CYP2E1 expression in centrilobular hepatocytes when compared to periportal hepatocytes. In our study, a decrease in the intensity of the CYP2E1 immunopositive signal in IHC-stained liver sections of rats subchronically exposed to AA indicates a decrease of CYP2E1 expression upon AA application. Our results are in agreement with El-Bohi et al. [28] who showed that CYP2E1 decreased at the protein and mRNA level in the livers of rats that were exposed to 50 mg/kg b.w. of acrylamide for 21 days. Contrarily, Singh et al. [37] observed an increased CYP2E1 expression in liver of mice treated with 50 mg/kg b.w. of AA for 5 days. Taken together, all these data may indicate that short, acute, AA treatment induces CYP2E1 expression, while prolonged, subchronic, AA exposure reduces CYP2E1 expression. A decrease of CYP2E1 expression was also observed in the liver of rats chronically exposed to ethanol [38]. It is suggested that downregulation of CYP2E1 could be a hepatoprotective response, since CYP2E1 stimulates oxidative stress and high levels of the enzyme are cytotoxic to liver cells [38]. In our experiment the significant AA-induced downregulation of CYP2E1 in animals treated with AA_25mg_ may represent a cytoprotective response, in order to decrease the formation of the more toxic glycidamide in hepatocytes. Reduction in CYP2E1 expression in animals exposed to AA_50mg_ was not prominent as in animals treated with AA_25mg_. The increasing toxicity of AA_50mg_ may overwhelm the hepatoprotective capacity i.e., the ability of hepatocytes to protect themselves from toxic effects. In line with in vivo findings, treatment with AA also reduced the CYP2E1 mRNA levels in the H4IIE cells. Our results obtained from both in vitro and in vivo models suggest that by downregulation of CYP2E1 expression, AA is able to affect its own metabolism in hepatocytes. Regulation of CYP2E1 by its own substrates has been reported previously, and in the liver it occurs at either the transcriptional or posttranscriptional level [39].

Nitric oxide (NO) is a very reactive oxidant formed in parenchymal and non-parenchymal liver cells [40]. It is produced as a by-product during the oxidation of L-arginine to citrulline in a reaction catalyzed by nitric oxide synthase (NOS) [41,42]. NO has an important role in liver physiology and pathophysiology [42]. Even though a moderate level of NO produced by iNOS is mostly beneficial, many disorders are caused by an overproduction of NO [40]. Our results are in line with Singh et al. [37], who observed an increase of NO levels and iNOS expression in the liver of mice treated with 50 mg/kg b.w. of AA. Besides hepatocytes, we observed iNOS expression in non-parenchymal liver cells. Activated Kupffer cells produce large amounts of NO via iNOS induction, and they are the main hepatic source of peroxynitrite [42]. An AA-induced increase of iNOS expression could imply that AA application could cause an increase of iNOS activity and that produced high concentrations of NO may lead to liver damage. An elevated amount of NO generated by iNOS is involved in the etiology of many hepatic disorders [42]. In line with our in vivo results, elevated iNOS transcription and nitrite levels in AA-treated H4IIE cells suggest the induction of both expression and activity of iNOS after AA treatment. An increase in iNOS expression and activity was also observed in AA-treated MCF-12-A and Rin-5F cells [29,43].

SOD and CAT play a pivotal role in maintaining cellular redox homeostasis [18]. SOD catalyzes the dismutation reaction of superoxide radicals to H_2_O_2_, which is then metabolized to H_2_O and O_2_ by CAT [44]. Hussain et al. [45] reported that an imbalance in antioxidant enzymes’ expression induces oxidative stress. Our in vivo results showed that an increased expression of SOD1 and SOD2 and an unchanged expression of CAT after AA application could indicate that AA promotes oxidative stress in the liver. Upon AA application, decreased SOD activity was detected in HepG2 cells [18,46,47] and rat liver [48,49]. In our study, we observed an increase in the expression, but a decrease in the activity, of SOD in H4IIE cells treated with AA, as reported previously for a rat pancreatic insulinoma cell line—Rin-5F [29]. Decreased CAT activity after AA administration was detected in HepG2 cells [18,46] and rat liver [48]. We observed increased CAT expression, while activity was unaffected in AA-treated H4IIE cells. Reduced (SOD) or unchanged (CAT) enzyme activity along with increased expression in AA-exposed H4IIE cells may be due to inactivation of excess protein that has been synthesized under conditions of high oxidative stress [50].

During detoxification of AA in the liver, most of AA is conjugated with glutathione and less is metabolized to GA [51]. GSH has a crucial role in neutralizing free radicals and reactive oxygen compounds [18]. We observed a decrease of GSH levels in the hepatoma H4IIE cell line after AA administration. These findings concur with previous studies which reported a decrease of GSH content upon AA application in isolated rat hepatocytes [52], the human hepatoblastoma cell line (HepG2) [47,52,53], and rat liver [48,54,55]. Exposure of H4IIE cells to 4.5 mM (IC_50_) AA elevated the total GST activity and the transcription of GSTA2 and GSTP1 genes. It has been reported that AA induces oxidative stress by reacting with GSH via GST and thus reduces its level in the cell [53]. A reaction between AA and GSH results in glutathione S-conjugates’ formation. Increased GST activity along with the increase of AA concentration may be a consequence of elevated production of S-conjugates between AA and GSH [52]. Our results are in agreement with previous studies showing an increase of GST activity upon AA application in isolated rat hepatocytes [52] and rat liver [56]. In our study, we observed increased lipid peroxidation in AA-treated-H4IIE cells, as reported previously for HepG2 cells [46,47,53] and rat liver [48,49,54,55,56]. Enhancement of lipid peroxidation could be a consequence of the depletion of glutathione to critical levels [29,56]. Although GSH content decreased in AA-exposed H4IIE cells, AA did not affect protein thiol groups’ level. Similar findings were reported previously for a rat pancreatic insulinoma cell line—Rin-5F [29]. It is suggested that in AA-treated cells, a reduced form of thioredoxin could interact with substrate proteins that contain a disulfide bond and reduce cysteine residues, and consequently inhibit the oxidation of cysteine [29,57]. Not including the effects of the AA toxic metabolite GA on hepatocytes is the limitation of the present study. Future in vitro and in vivo studies, besides AA exposure, should include GA exposure in order to elucidate the mechanisms of AA action. In addition, AA application to CYP2E1 knock-out cells should further clarify the mechanism of AA toxicity.

## 5. Conclusions

In summary, we conclude that AA treatment affects the redox balance and CYP2E1 expression in H4IIE cells. In addition, in the liver of rats subchronically treated with AA, increases of iNOS, SOD1, and SOD2, and a decrease of CYP2E1 expression was detected. Obtained results indicate that AA, by changing hepatocytes drug-metabolizing potential and disturbing its redox status, exerts potential hepatotoxic effect.

## Data Availability

All relevant data are available from the corresponding author on request (jelena.markovic@dbe.uns.ac.rs).
